# Modulation of Motor Cortex Activity After Intrathecal Baclofen Delivery in Chronic Thoracic Spinal Cord Injury

**DOI:** 10.3389/fneur.2022.778697

**Published:** 2022-05-13

**Authors:** Ivana Štětkářová, Jiří Keller

**Affiliations:** ^1^Department of Neurology, Third Faculty of Medicine, Královské Vinohrady University Hospital, Prague, Czechia; ^2^Department of Radiology, Na Homolce Hospital, Prague, Czechia

**Keywords:** intrathecal baclofen, functional MRI, plasticity, motor cortex activity, spinal cord injury

## Abstract

**Objectives:**

Intrathecal baclofen (ITB) is commonly used for reduction of spasticity in chronic spinal cord injury (SCI). Its clinical effect is well-known; however, exact mechanisms of long-term effect of continuous ITB administration (cITBa) on modulation of cortical processes have not been elucidated. The aim of this study was to evaluate changes in motor cortex activation for healthy upper limbs in comparison to impaired lower limbs by functional magnetic resonance imaging (fMRI).

**Methods:**

Ten subjects (eight males, 20–69 years) with thoracic SCI presenting no voluntary movements of lower limbs (except one) were enrolled in the fMRI study. fMRI at 1.5T with a finger tapping paradigm and mental movement simulating foot flexion on the dominant side were performed before, 3 months, and 1 year after start of cITBa. fMRI data processing was carried out using FMRI Expert Analysis Tool (FEAT), part of FSL. A second-level analysis was carried out using FLAME stages 1 and 2. The level of spasticity was assessed with the Modified Ashworth scale (MAS).

**Results:**

Continuous ITB significantly decreased limb spasticity in all the subjects (group MAS spasticity dropped from 3 to 0.3). The second-level analysis (Z > 1.6, cluster significance threshold *p* =0.05) revealed increased activation of the primary sensorimotor cortex of the foot between baseline and 3 months, and 3 months and 1 year.

**Conclusion:**

Increased sensorimotor cortex activation with spasticity reduction after cITBa may reflect distant functional reorganization because of long-term mediated neuroplastic changes in the sensorimotor cortex. Better understanding of modulation of brain function in SCI after cITBa may influence the field of neurorehabilitation.

## Introduction

Spasticity is a severe clinical manifestation of upper motor neuron lesions. It has been reported in 10–65% of patients with chronic spinal cord injury (SCI) (1–4). Neural connectivity after SCI is altered in the spinal cord; however, reorganization of the brain is considered to be an important mechanism following SCI, requiring adaptive changes in surviving circuitries.

Spasticity in subjects with SCI may be alleviated by non-pharmacological treatment (5), and pharmacotherapy is the most common treatment (6–8). A wide variety of drugs is recommended, with different action sites, but the most widely used medicine is baclofen, a gamma-aminobutyric acid (GABA B) receptor agonist. A very good effect of continuous intrathecal baclofen (cITB) administration using pump delivery systems has been demonstrated in generalized SCI spasticity (9–12); however, associated complications influence its utility and acceptance (13–17). Intrathecal baclofen delivery concentrates the drug in the cerebrospinal fluid at higher levels, and patients can use concentrations of baclofen <100 of those used orally. Thus, the central side effects of oral baclofen, such as drowsiness, fatigue, and difficulties to concentrate, appear to be minimized with intrathecal administration. Moreover, long-term intrathecal baclofen delivery, even at a high dose, has only a minor influence on cognitive functions (18).

To this date, little is known about pathophysiological mechanisms involved in spinal spasticity after neuromodulatory treatment as cITB delivery. ITB is considered to act differently at the spinal, brainstem, and cortical levels (19, 20). The impact of neuronal inhibition on the functional activity of the brain has already been studied with baclofen as a GABA(B) receptor agonist generally decreasing metabolic activity (21). Increased activation of the sensorimotor cortex has been observed by functional magnetic resonance imaging (fMRI) 1 month after baclofen pump implantation in one patient with multiple sclerosis (22). Sensorimotor network changes after chronic SCI treated by intrathecal baclofen delivery has not yet been systematically studied.

The aim of this study is to determine by fMRI whether long-term administration of cITB has any effect on cortical plasticity changes over time in patients with SCI. We studied a selected population of patients with chronic thoracic SCI and compared fMRI data prior to baclofen pump implantation, 3 months after, and 1 year after cITB pump delivery.

## Methods

From our database, we selected 15 patients with chronic SCI who fulfill the criteria for ITB pump implantation, e.g., low response to oral antispastic treatment and severe limb spasticity of 3 or 4 according to the Modified Ashworth scale (MAS), who underwent a positive clinical trial with ITB administration through a temporarily inserted intrathecal catheter. For further analysis to keep the homogeneity of lesion localization, we finally selected 10 subjects (eight males, aged 19–69 years, mean of 46) with only thoracic SCI. We excluded patients with traumatic brain injury as well as patients with peripheral nerve lesions or any neurological condition influencing the brain or spine (e.g., multiple sclerosis). Therefore, thoracic spine lesion is, in our cohort, the only neurological and neuroradiological condition that impairs motor functions. To achieve this selection, all the subjects underwent brain and spinal cord magnetic resonance imaging (MRI) before cITB pump implantation, and diagnostic brain MRI was performed in each fMRI session.

## Neurological Evaluation

All the subjects were neurologically examined by an experienced neurologist (IS) in terms of motor, sensory, and cognitive functions before, and 3 months and 1 year after cITB pump implantation. The clinical testing of patients used standard scales, the AIS Scale (American Association of Spinal Cord injury Impairment Scale) of spinal cord function after injury (23) and the MAS of muscle hypertonia (grades 0–4) (24). The MAS is the most accepted clinical tool used to measure the level of muscle tone (25).

In this study, evaluation of spasticity using MAS was performed every visit before and 3 months and 1 year after cITB pump implantation. This evaluation was performed again by the experienced neurologist (IS). The MAS was assessed separately for dorsal and plantar flexions of the foot and flexion and extension of the knees and hip. The values were averaged and calculated as mean MAS for each subject. The daily dose of baclofen was assessed every visit according to the level of spasticity. The patients were not under supervision with specific rehabilitation; they exercised at home and performed their normal daily activities.

## Functional Magnetic Resonance Imaging

All the subjects have also been examined with fMRI techniques using a detailed protocol of motor functions. All the patients were informed, in a comprehensible manner, about the risks arising from fMRI and the possibility to withdraw from the study at any time. Pump function was tested within 30 min after the completion of the fMRI procedure to avoid any complication, e.g., unsuccessful restart of the function after a possible brief stop of pumping during the examination. To further limit potential risks from the MRI examination, a 1.5 T scanner (Siemens Avanto, Erlangen, Germany) equipped with quantum gradient coils and a standard head coil) was used (lower static magnetic field induces lower force to the device compared to 3T).

Two motor tasks were employed: finger-tapping and foot flexion were studied by 1.5T fMRI with movement on the dominant side (the data of two left-handed subjects was flipped in the x axis to have the activation on the same side of the image).

The tasks were performed before and 3 months and 1 year after cITB pump implantation. The patients were scanned while performing two tasks: (1) imaginary movement of impaired foot and (2) real finger movement with the healthy hand. All the subjects had preserved finger movements and performed sequential finger movements at a rate of approximately 1 movement per second. In the second task, the subjects were asked to imagine performing the movement of repetitive dorsal and plantar flexions at the same frequency as for the hand finger movements.

In both tasks, a block paradigm was used (TR = 3,000 ms, TR = 51 ms, voxel size 3 mm isotropic): after two dummy pulses, 16 volumes (48 s) of rest data and 16 volumes (48 s) of task data were repeatedly acquired. A total of four pairs of conditions were performed for a total acquisition length of each fMRI experiment of 6.5 min. Inside the bore of the scanner, the task was performed with the eyes open, and instructions were given with a color square in the middle of the visual field, red to stop and green to start the task. While performing the active movement task, the subjects were carefully observed to follow the pattern of the design (in an imaginary task, no such check is possible).

Standard morphological examinations (T1- and T2-weighted imaging, FLAIR) were further performed to exclude brain pathology.

FMRI data processing was carried out using FMRI Expert Analysis Tool (FEAT) Version 6.00, part of FSL (FMRIB Software Library, www.fmrib.ox.ac.uk/fsl). In the first-level analysis, two dummy pulses were removed, temporal high-pass filter was 90 s, and brain extraction and 6-mm spatial smoothing were applied. Motion correction was performed using MCFLIRT. A statistical analysis for this step was performed using FILM prewhitening and both standard and extended motion parameters. The second-level analysis was carried out using FLAME stages 1 and 2, and Z (Gaussianized T/F) statistic images were thresholded using clusters determined with Z > 1.6 and a (corrected) cluster significance threshold of *P* =0.05 (26, 27).

## Results

All the patients had SCI because of a fall or a motor vehicle accident and were immediately operated after SCI; only one patient (no. 6) suffered from spinal arterial-venous malformation (AVM) and he underwent embolization of AVM twice. This subject was included, as the level of lesion was identical to that in the other subjects; however, his loss of motor function was incomplete (AIS C). All the remaining subjects had paraplegia (AIS A) with loss of motor function. Detailed clinical characteristics of the patient group are summarized in Table 1.

**Table 1:** Demographic and clinical characteristics of thoracic spinal cord injury patients.

**No**.	**Sex**	**Age**	**Etiology**	**Injury (years)**	**Level of lesion**	**AIS**	**Level of sensory loss**	**MAS before impl**.	**MAS 3 months after impl**.	**MAS 1 year after impl**.	**ITB dose 3 months after impl**.	**ITB dose 1 year after impl**.
1*	M	38	Fall from tree	1,5	T2	A	T2	3	0	0	165	270
2	M	42	Motor vehicle accident	3	T3	A	T5	2,5	0	0	225	400
3	F	19	Fall from wall	3	T4	A	T5	4	1,5	1	120	160
4	M	50	Fall from roof	18	T4	A	T4	3	1	1	110	125
5	M	45	Motor vehicle accident	18,5	T5-6	A	T5	3	1	0	95	180
6	M	45	Spine AVM	3	T6-7	C	T8	4	1	1	300	390
7	M	48	Motor vehicle accident	5,5	T7	A	T7	3,5	1	0	90	150
8	M	64	Fall from roof	1,5	T7	A	T7	3	2	0	92	150
9*	M	44	Motor vehicle accident	2	T7-8	A	T8	2	0	0	125	160
10	F	69	Fall from ladder	4	T8	A	T9	2	0	0	100	170

Note: *left-handed.

*T, thoracic; impl., implantation; MAS, Modified Ashworth Scale; AIS, American Spinal Cord Injury Association Impairment Scale; ITB, intrathecal baclofen, dose in ug/day*.

The level of limb spasticity assessed by the Modified Ashworth scale (MAS) significantly decreased in all the subjects, whereas group MAS limb spasticity dropped from 3 to 0.75 after 3 months of cITB treatment, and to 0.3 1 year after cITB pump implantation. All the patients had continuous baclofen administration, with an average baclofen dose of 142.2 μg/day (range 90–300 μg/day) 3 months after cITB pump implantation and 215.5 μg/day (range 125–400 μg/day) 1 year after cITB pump implantation. During the whole study, no ITB drug side effects (e.g., overdose, excessive weakness, drowsiness, and withdrawal syndrome), complications resulting from pump implantation, and failure of the pump system, as well as complications during standard baclofen filling of the reservoir were observed.

Diagnostic brain MRI was performed every fMRI session and read as negative by an experienced neuroradiologist (JK).

**Figure 1:**
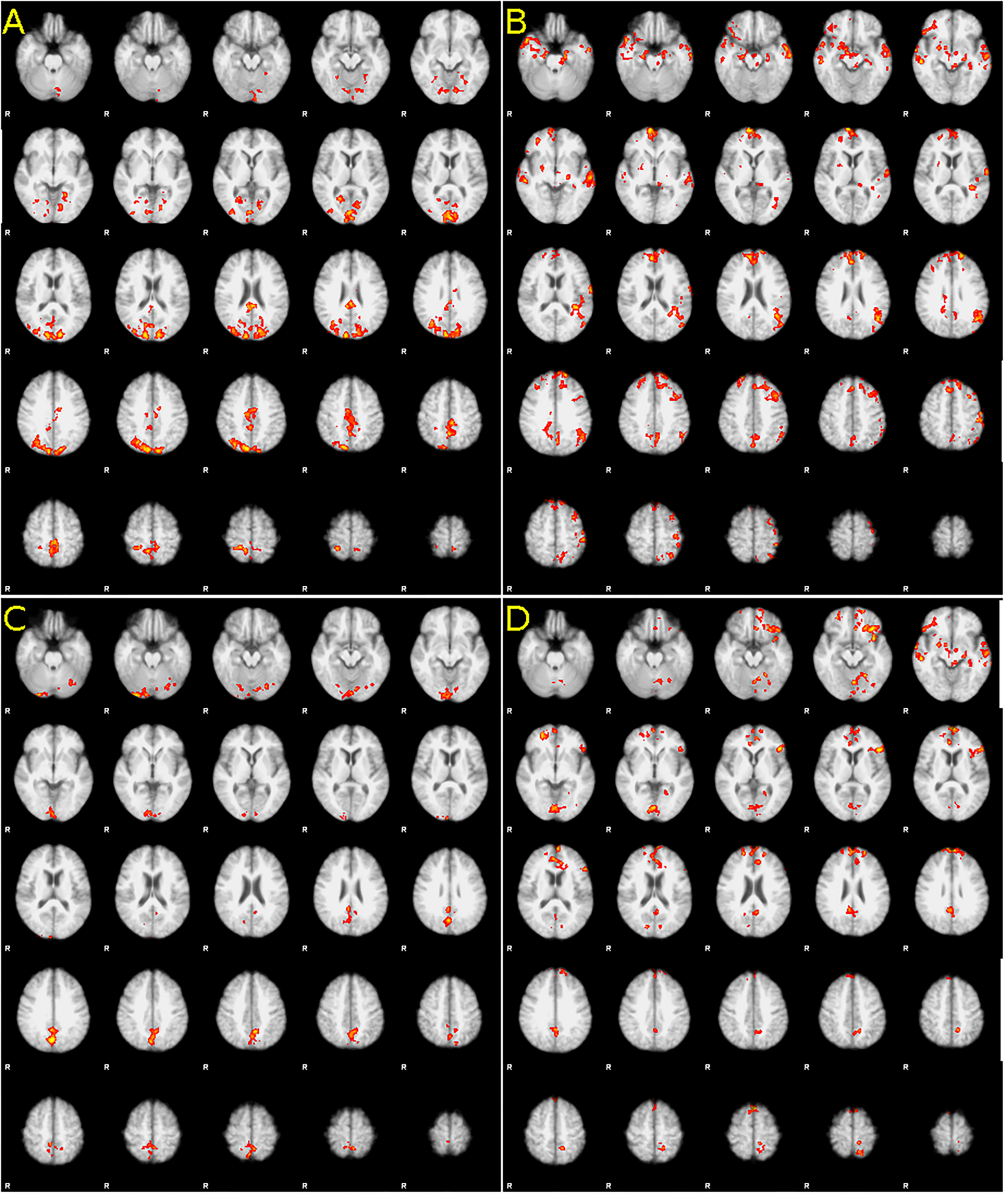
Difference in activation between [**(A,C**), left column) baseline and 3 months after baclofen pump implantation, and [**(B,D)**, right column] 3 and 12 months after implantation. Increase in foot in both, **(A)** baseline and 3 months comparison and **(B)** 3 and 12 months, **(C)** Increase in hand in the first 3 months, and decrease (!) **(D)** between 3 and 12 months. Statistic images were thresholded non-parametrically using clusters determined with Z > 1.6 and a (corrected) cluster significance threshold of *P* = 0.05. For full statistical output, please see Supplementary Materials.

After the first-level statistical analysis (see Methods above), the second-level analysis was performed on the data from both tasks, comparing pairwise baseline to 3 months and 3 months to 12 months: in the mental foot flexion task, an increase in fMRI activation was detected in the comparison between pre-implant and 3 months of follow-up, as well as between 3 and 12 months of examination (Figure 1), predominantly in the bilateral primary motor areas. Between baseline and 3 months, the increase is not only visible in the primary motor area but also in the parietal lobes, sensorimotor area, parts of occipital regions, and in a small cluster in the left cerebellar hemisphere. Between 3 and 12 months, the activation increased again in the primary motor cortex and in the bilateral frontopolar and temporobasal regions.

In the real motor finger tapping task 3 months after pump implantation, an increase in activation was present in the apical part of both cerebellar hemispheres (more on the right) and in the central region (more on the left). Between months 3 and 12, a nearly identical image is seen in an inverted manner: there is a statistically significant decrease in activation (confirmed by testing baseline-12 months with no difference, data not shown). In upper limb, an increase between the baseline and month 3 was detected when MAS was used as a primary measure, including the order of the examination as a confound factor (see Supplementary Material).

## Discussion

Continuous intrathecal baclofen administration (cITBa) profoundly relieving spasticity in patients with chronic thoracic SCI was associated with increased activation of the sensorimotor cortex of plegic legs 3 months and 1 year after cITBa, which may reflect distant functional reorganization of the sensorimotor network at the cortical level. The activation observed in the primary motor areas, parietal lobes, sensorimotor area, parts of occipital regions and cerebellum may help to compensate for functional deficit following SCI.

Recovery of function following injury in the nervous system requires adaptive changes in surviving circuitries. Neural connectivity has not only been shown to be altered after SCI in the spinal cord but also in the brain (28, 29). Resting-state fMRI in animal studies (30) after complete thoracic SCI showed that the functional connectivity between the primary motor and primary sensory areas was significantly decreased, and that the connectivity between the primary motor and motivation areas was increased and, thus, explained as a time-dependent compensatory upregulation of “motor functional motivation”. Motor and sensory deficits after SCI result in functional reorganization of the sensorimotor network (28, 31), which has also been documented by several task-evoked functional magnetic resonance imaging human studies (32). In a meta-analysis of fMRI studies, reorganization primarily occurred in the sensorimotor system of the brain after SCI, implying that brain functions involved in sensorimotor demands can still be preserved in this condition (33). In addition, a whole-brain meta-analysis revealed increased activation in the cerebellum, and this increase was positively correlated with lesion level and injury severity.

In a longitudinal study on patients with quadriplegia (32), a gradual reduction in the extent of activation in the primary motor cortex was detected, which was accompanied by a transient increase in the activity of sensorimotor areas. Nakanishi (34) recently investigated 8 subjects with chronic SCI by fMRI and found that the subjects with SCI showed higher grip force steadiness, smaller activation in the primary motor cortex, and deactivation of the visual cortex. Min et al. (35) performed resting-state fMRI and presented opposing findings of increased functional connectivity between the primary motor cortex and other motor areas. They concluded that motor components in the motor network increased in functional connectivity in order to compensate for motor deficits. In our study, patients with SCI and plegic lower limbs showed increased activity in the primary motor cortex after ITB delivery, and this observation was accompanied with wider activation in the sensorimotor area, parietal lobes, visual cortex, and cerebellum. One explanation for this observation may be that long-term sensory deprivation due to SCI increases cortical excitability and accelerates the plasticity of other brain areas, but therapeutic-mediated mechanisms may also play an important role in functional restoration (36).

Patients with chronic SCI in the thoracic region have more activated areas in the somatosensory cortex for the healthy upper limbs (29). Motor function after the injury may be enhanced, as individuals with SCI need their remaining body parts to compensate for lost function and to adapt their daily life with their remaining intact limbs (34). A similar observation was also made in this study with the real motor finger tapping task. Three months after ITB pump implantation, an increase in activation was present in the central region. One year after ITB pump implantation, we observed functional “normalization” with a decrease in activation in this region. A possible explanation may be the good clinical response to ITB treatment, with the overall relief of leg spasticity inducing adaptive mechanisms in brain and spinal cord connections. However, the observed brain reorganization could be not only due to pharmacotherapy and decrease in spasticity but also natural compensatory neuroplastic changes. These changes may also depend on the level of rehabilitation and individual activities of daily living. Similarly, it can be observed with botulinum toxin treatment for focal spasticity, where botulinum toxin becomes gradually accepted as a promising tool to correct maladaptive plastic changes in the sensorimotor cortex in stroke and dystonia (37, 38).

In the last few years, an increasing number of studies have also presented the cerebellum as another important subcortical brain structure in patients with chronic SCI (31, 33, 39). Chen (40) performed sensory-task related fMRI on subjects with incomplete SCI and documented the existence of an alternative pathway in the impairment of somatosensory function after SCI, which consists of the ipsilateral cerebellum, brainstem, and contralateral postcentral gyrus. In our study, we also found a significant increase in fingers movement-induced fMRI activation of several brain areas, especially in sensorimotor control and motor learning. Our data support that the successive increase in activation in mental tasks may reflect a functional reorganization of the motor cortex involved in impaired leg function. The increase in activation during the first examination 3 months after the implantation may be explained by the co-activation of lower-limb motor areas and the cerebellum in the early adaptation stage, which returns to normal after stabilizing the daily dosage of baclofen.

GABAergic pathways are involved in the regulation of muscle tone. In chronic SCI, there is a decrease in GABA, the inhibitory neurotransmitter involved in presynaptic inhibition (41). As we have already known, loss of supraspinal control and GABA dysfunction in the spinal cord are some of the pathophysiological mechanisms involved in spasticity (42); thus, it may be possible that the continuous administration of baclofen (GABA analog) modulating the spasticity in our patients with SCI contributes, to some extent, to the observed motor cortical changes.

## Conclusion

We conclude that long-term ITB administration relieves spasticity and results in various changes in the morphological substrate of neural networks with distant functional reorganization of the sensorimotor network at the cortical level due to long-lasting neuroplastic changes. We are currently unable to determine what plays a major role in above mentioned long-term brain reorganization if pharmacotherapy with decrease of spasticity, GABA modulation, the natural course of the disease or combination of all mechanisms.

Detailed understanding of the mechanisms of corticospinal plasticity in motor learning will help to improve restoration of motor functions after spinal cord injury and may have broader applications in the field of neurorehabilitation.

## Clinical/Research Implications and Future Directions

This study has implications for clinical outcomes of patients with SCI, demonstrating how continuous baclofen delivery relieves spasticity in these subjects together with long-term modulation of motor cortex activity. Better knowledge of this motor cortex activation/inhibition may help in rehabilitation/restoration of the sensorimotor network after chronic spinal cord lesions. Future studies are needed to understand how the natural course of chronic SCI develops by predicting clinical presentations of severe spasticity responding to a variety of personalized therapeutic approaches, including pharmacological treatment such as intrathecal baclofen delivery.

## Study Limitations

Due to logistic and ethical barriers in patient recruitment including overall cost and proximity to MRI facilities and cITB treatment, we included only treated cITB group. We tried to get the group homogeneity as much as possible in terms of localization of the thoracic spinal cord lesion and similar clinical picture with lower extremities impairment. Thus, only one subject was able to have a voluntary movement in his lower limbs. He performed the same “mental” tests as the others. The small sample size of our group limits the application of these findings to the broader SCI population.

Future studies involving a representative sample of cITB and non-cITB patients as well as patients treated with a different drug for spasticity are needed to further validate motor cortex activation in terms of natural course of chronic SCI and/or baclofen-specific effect.

## Data Availability Statement

The raw data supporting the conclusions of this article will be made available by the authors, without undue reservation.

## Ethics Statement

This study involving human participants were reviewed and approved by the Local Ethical Committee of University Hospital (Ethical Committee of Královské Vinohrady University Hospital, No. EK-VP/16/0/2016). All subjects provided their written informed consent to participate in this study and were informed of the possibility to withdraw from the study at any time.

## Author Contributions

IŠ and JK had a major role in the conceptualization and design of the study, data analyses, interpretation of the findings, and writing of the manuscript. JK also conducted functional magnetic resonance data assessment. Both authors contributed to the article and approved the submitted version.

## Funding

This research was supported by the program Cooperation - Neuroscience, Charles University, and by a grant from the Ministry of Health of the Czech Republic-DRO (NHH, 00023884).

## Conflict of Interest

The authors declare that the research was conducted in the absence of any commercial or financial relationships that could be construed as a potential conflict of interest.

## Publisher's Note

All claims expressed in this article are solely those of the authors and do not necessarily represent those of their affiliated organizations, or those of the publisher, the editors and the reviewers. Any product that may be evaluated in this article, or claim that may be made by its manufacturer, is not guaranteed or endorsed by the publisher.

## Supplementary Material

All Z (Gaussianized T/F) statistic images were thresholded non-parametrically using clusters determined with Z > 1.6 and a (corrected) cluster significance threshold of *P* = 0.05.

Supplementary Figure S1 All slices from Figure 1 (see Figure 1 legend for details).

Supplementary Figure S2 Task for the upper limb, comparison between baseline and 3-month follow-up studies. Areas where activation is higher have higher Modified Ashworth scale (MAS) values, order of the examination is used as a confound factor.

Supplementary Figure S3 Task for the upper limb, comparison between 3- and 12-month follow-up studies. Areas where activation is higher have higher MAS values, order of the examination is used as a confound factor.
