# Supplementary figures and images for: Modulation of Motor Cortex Activity After Intrathecal Baclofen Delivery in Chronic Thoracic Spinal Cord Injury
Source: Front Neurol. 2022 May 13;13:778697. doi: 10.3389/fneur.2022.778697 (PMC9136289; doi:10.3389/fneur.2022.778697)

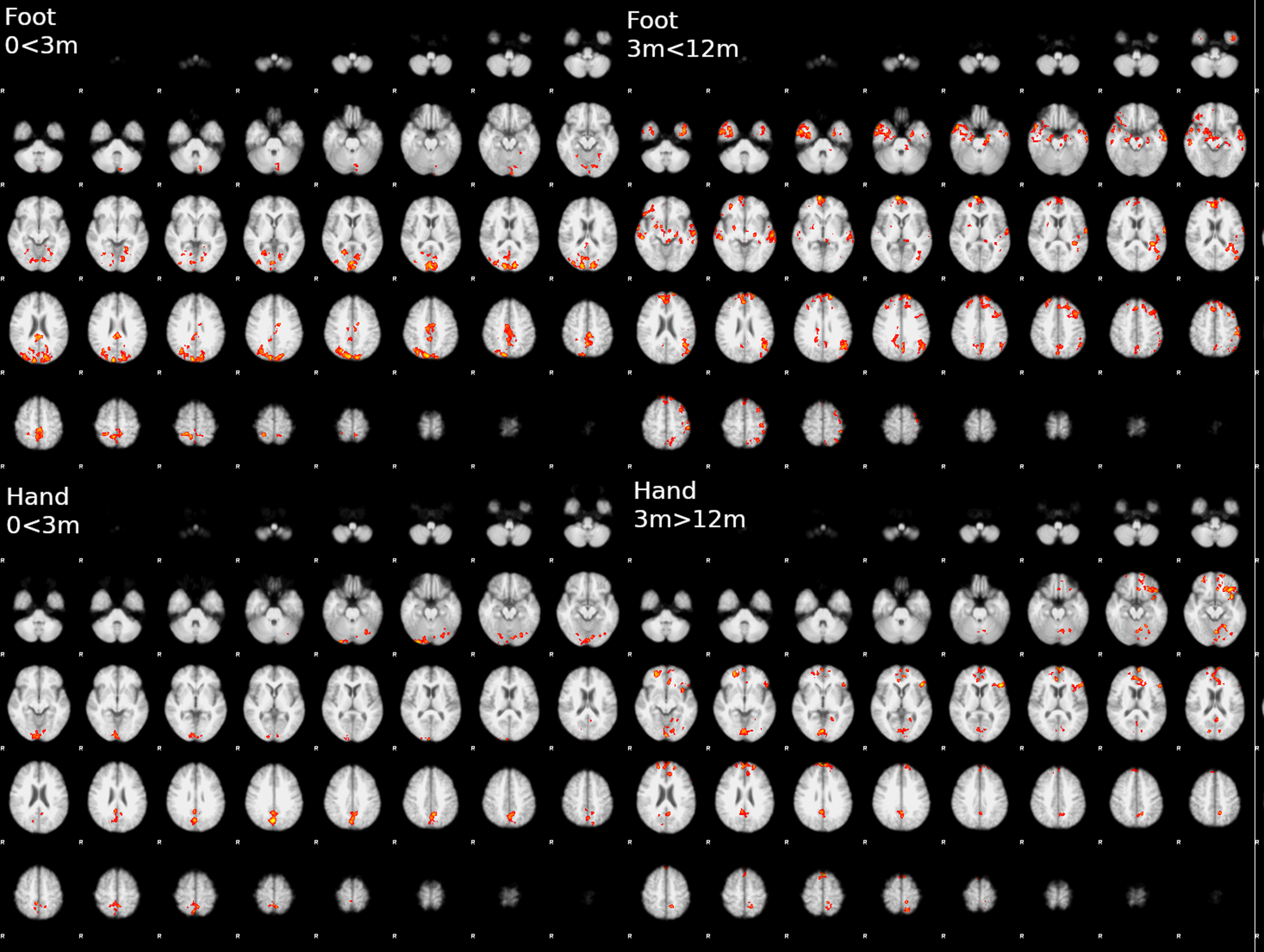

Supplement: Supplementary Figure S1 — All slices from Figure 1 (see Figure 1 legend for details). [file Image_1.TIFF]

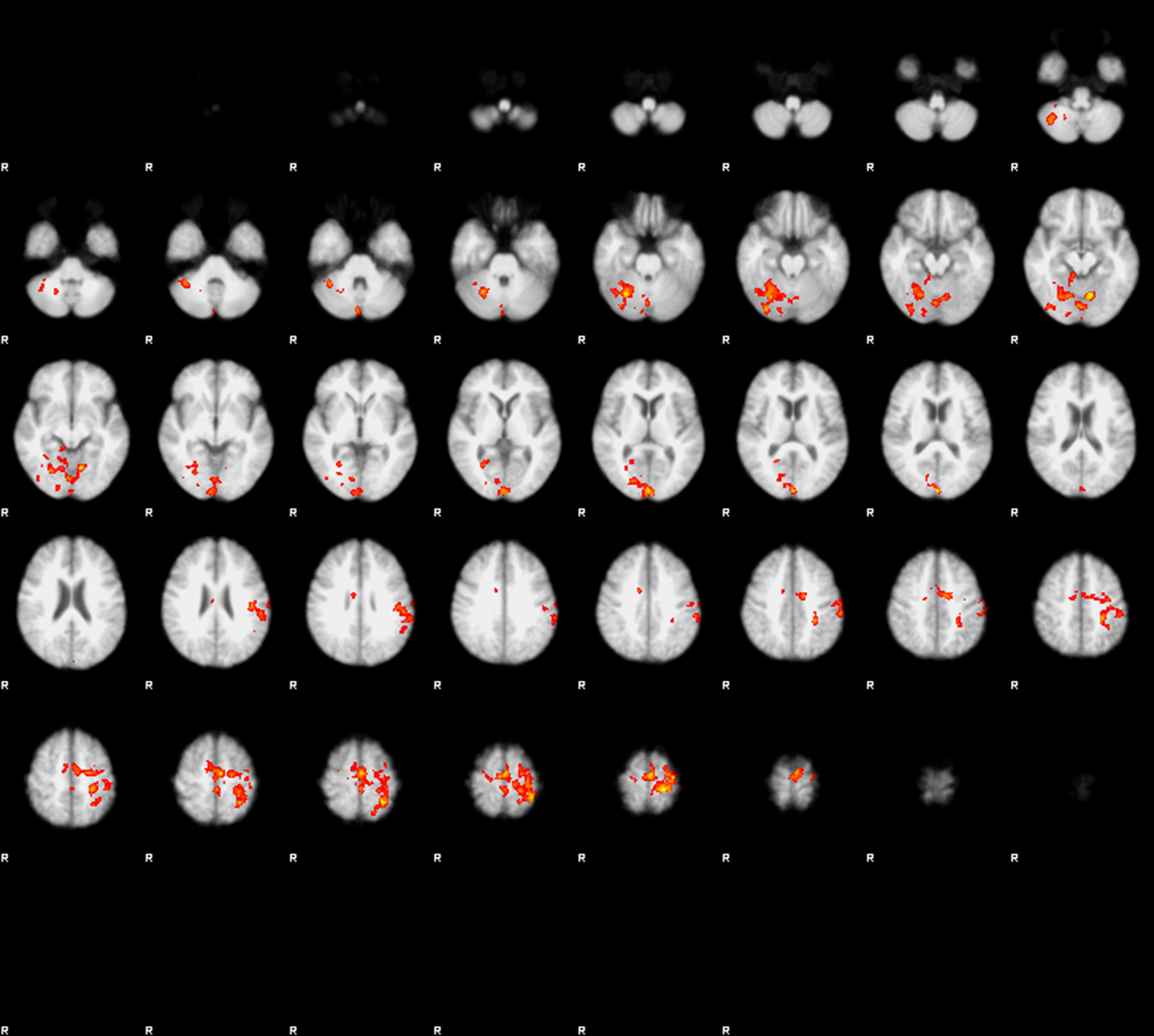

Supplement: Supplementary Figure S2 — Task for the upper limb, comparison between baseline and 3-month follow-up studies. Areas where activation is higher have higher Modified Ashworth scale (MAS) values, order of the examination is used as a confound factor. [file Image_2.TIFF]

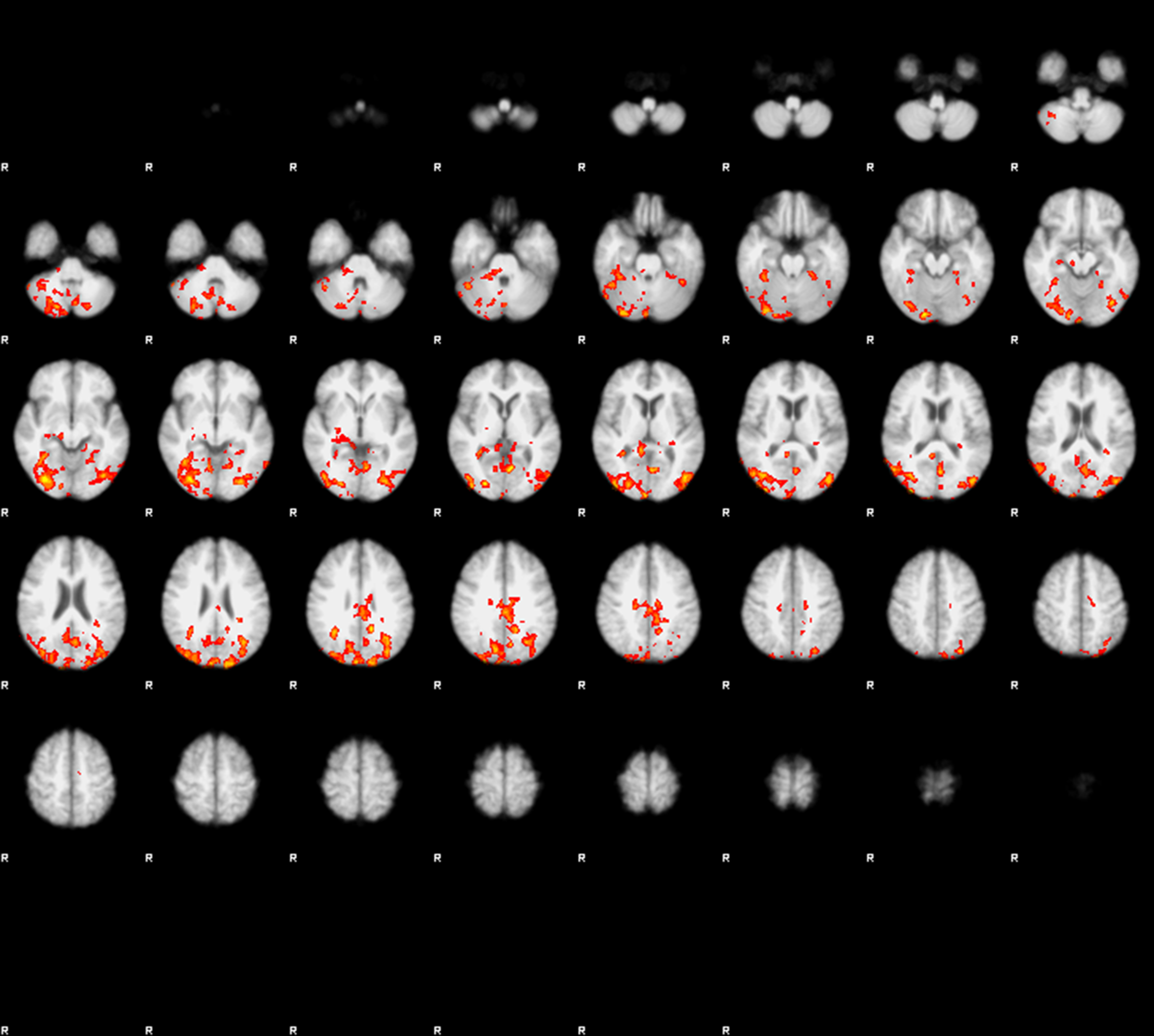

Supplement: Supplementary Figure S3 — Task for the upper limb, comparison between 3- and 12-month follow-up studies. Areas where activation is higher have higher MAS values, order of the examination is used as a confound factor. [file Image_3.TIFF]
